# Correlation of *DAPK1* methylation and the risk of gastrointestinal cancer: A systematic review and meta-analysis

**DOI:** 10.1371/journal.pone.0184959

**Published:** 2017-09-21

**Authors:** Wenzheng Yuan, Jinhuang Chen, Yan Shu, Sanguang Liu, Liang Wu, Jintong Ji, Zhengyi Liu, Qiang Tang, Zili Zhou, Yifeng Cheng, Bin Jiang, Xiaogang Shu

**Affiliations:** 1 Department of Gastrointestinal Surgery, Union Hospital, Tongji Medical College, Huazhong University of Science and Technology, Wuhan, China; 2 Department of Emergency Surgery, Union Hospital, Tongji Medical College, Huazhong University of Science and Technology, Wuhan, China; 3 College of Clinical Medicine, Hubei University of Science and Technology, Xianning, China; 4 Department of Hepatobiliary Surgery, The Second Hospital, Hebei Medical University, Shijiazhuang, China; 5 Department Breast & Thyroid Surgery, TongJi Hospital, Tongji Medical College, Huazhong University of Science and Technology, Wuhan, China; University Hospital Llandough, UNITED KINGDOM

## Abstract

**Objective:**

One of the critical mechanisms of gastrointestinal cancer pathogenesis is the silencing of death associated protein kinase 1 (*DAPK1*), which could be caused by aberrant methylation of the promoter. However, the relationship between *DAPK1* methylation and the risk of gastrointestinal cancer is still controversial. Hence, we conducted this study to determine the potential correlation.

**Methods:**

Eligible publications were searched in the Pubmed, Embase, and Cochrane Library through November 2016 according to the inclusion criteria and exclusion criteria. Revman 5.3 and Stata 12.0 software were used to analyze the relevant data regarding the association between the frequency of *DAPK1* methylation and gastrointestinal cancer.

**Results:**

A total of 22 studies with 2406 patients were included in this meta analysis. Methylation of *DAPK1* was positively related with the risk of gastrointestinal cancer (odds ratio [OR] = 5.35, 95% confidence interval [CI]: 2.76–10.38, P<0.00001, random effects model). The source of heterogeneity was analyzed by sensitivity analysis and subgroup analysis. After omitting one heterogeneous study, the I^2^ decreased and the OR increased in pooled analysis. Also, the heterogeneity decreased most significantly in the subgroup of studies that had a sample size of less than 60 cases. Then, the correlations between *DAPK1* methylation and clinicopathological features of gastrointestinal cancer were assessed. *DAPK1* methylation was positively correlated with the lymph node (N) stage (positive vs. negative, OR = 1.45, 95%CI: 1.01–2.06, P = 0.04, fixed effects model) and poor differentiation (OR = 1.55, 95%CI: 1.02–2.35, P = 0.04, fixed effects model) in gastric cancer, and the association was significant among Asian patients. However, among cases of gastrointestinal cancer, the association between *DAPK1* methylation and tumor (T) stage, N stage, distant metastasis (M) stage, and cancer differentiation were not statistically significant.

**Conclusions:**

*DAPK1* methylation is a potential biomarker for the early diagnosis of gastrointestinal cancer. Further analysis of the clinicopathological features indicated that aberrant methylation of DAPK1 is positively associated with the tumorigenesis of gastrointestinal cancer, and metastasis of gastric cancer.

## Introduction

Despite advances in the treatment of gastrointestinal cancer, it is still the leading cause of cancer-related mortality. For instance, gastric cancer (GC) ranks second, colorectal cancer (CRC) ranks fourth, and esophageal cancer (EC) ranks sixth as the most deadly cancers globally[1]. Increasing numbers of studies have been performed to demonstrate the mechanism of carcinogenesis, and to identify biomarkers for early diagnosis of gastrointestinal cancer[2].

Methylation of DNA is dramatically altered in cancers. Promoter CpG islands methylation is one type of DNA methylation that could result in the inactivation of tumor suppressor genes[3], such as *death-associated protein kinase 1* (*DAPK1*). *DAPK1* is a member of the Ser/Thr kinase family, and was found originally in interferon gamma (INF-γ)–induced death in HeLa cells [4]. Its critical role in regulating cell death and autophagy has been demonstrated[5]. In addition, *DAPK1* could be involved in multiple cell death processes induced by a variety of internal and external apoptotic stimulants, such as tumor necrosis factor-alpha and Fas ligand, and could mediate the pro-apoptotic pathway[6].

As a well-known tumor suppressor gene, *DAPK1* expression can suppress tumor growth and metastasis[7]. It has been confirmed that *DAPK1* is epigenetically silenced through methylation of its promoter in various human cancers including gastrointestinal cancer[8–10]. However, it remains controversial whether *DAPK1* promoter methylation is related to the risk of gastrointestinal cancer. Previous studies have reported that the *DAPK1* promoter methylation is much more frequent in EC, GC, CRC cancer tissues than that in control tissue[8, 10–12]. However, in some other studies, the frequency of *DAPK1* methylation showed no obvious increase[13] or even a reverse trend[14] in cancer samples. Therefore, we conducted this meta analysis to investigate the correlativity between *DAPK1* promoter methylation and gastrointestinal cancer.

## Methods

### Search strategy

We searched the Pubmed, Embase, and Cochrane Library electronic databases to, find the eligible articles using the search terms “*DAPK1*”, “death-associated protein kinase 1”, “DAPK”, “DAP kinase”, or “DAPK protein” with “neoplasms”, “cancer”, “tumor”, or “neoplasia” through November04, 2016. We also searched the reference lists of relevant articles to find additional qualified articles. Only publications written in English were selected. Among all the articles that we had searched, unrelated studies were excluded by reading the title and abstract. Then, full texts of the candidate studies were inspected thoroughly to determine whether they met the inclusion and exclusion criteria.

The inclusion criteria were as follows: 1. studies that evaluated the association between *DAPK1* methylation and gastrointestinal cancer, including EC, GC and CRC; 2. diagnosis of gastrointestinal cancer was histologically confirmed; 3. methylation status was examined by methylation-specific polymerase chain reaction (MSP); and 4. definitive data for the frequency of *DAPK1* methylation were provided.

We excluded unsuitable studies according to the following criteria: 1. the studies were performed without a control group; 2. the cancer group included cases of diverse precancerous lesions; 3. peripheral blood or other non-epithelium tissue was used as the object of detection; and 4. data regarding the frequency of *DAPK1* methylation could not be extracted from the raw data.

The quality of the included studies was assessed on the basis of the Newcastle-Ottawa Quality Assessment Scale (NOS). Four stars were used to evaluate the selection of study groups. Two stars were used to estimate the comparability of cases and controls. and three stars were used to value the exposure. Publications that scored less than 6 stars were excluded[15].

### Data extraction

Data in the text, figures, and tables of included studies were extracted by two authors using a data collection form that included author names, publication year, country, geographic area, method for detecting DNA methylation, source of the control group, number of patients, age distribution, gender distribution, and clinicopathological features (tumor stage, lymph node stage, distant metastasis and differentiation), follow-up time, and 5-year overall survival (OS) and disease-free survival (DFS) rates. The GetData Graph Digitizer v2.24 was used to extract the data from figures[16]. Discussions were held by three authors when uncertainty was encountered in data extraction.

### Statistical analysis

Review Manager 5.3 and Stata 12.0 software were used to analyze the data. Forest plots were generated to analyze the ORs and 95%CIs. Heterogeneity among studies was assessed by Q and I^2^ tests. An I^2^ value of 0% indicates no observed heterogeneity, whereas, 25% indicates low, 50% indicates moderate and 75% indicates high heterogeneity[17]. A random effects model was utilized when the heterogeneity is high, otherwise, the fixed effects model was applied. Sensitivity analysis and subgroup analysis were conducted to find the potential source of heterogeneity. Publication bias was qualitatively assessed by funnel plot generation which was conducted using Revman 5.3, and quantitatively evaluated by Egger weighted regression test and Begg rank correlation test, which were calculated using Stata 12.0 software. A P value ≤0.05 was regarded as statistically significant.

## Results

### Inclusion of studies in meta-analysis

**Fig 1:**
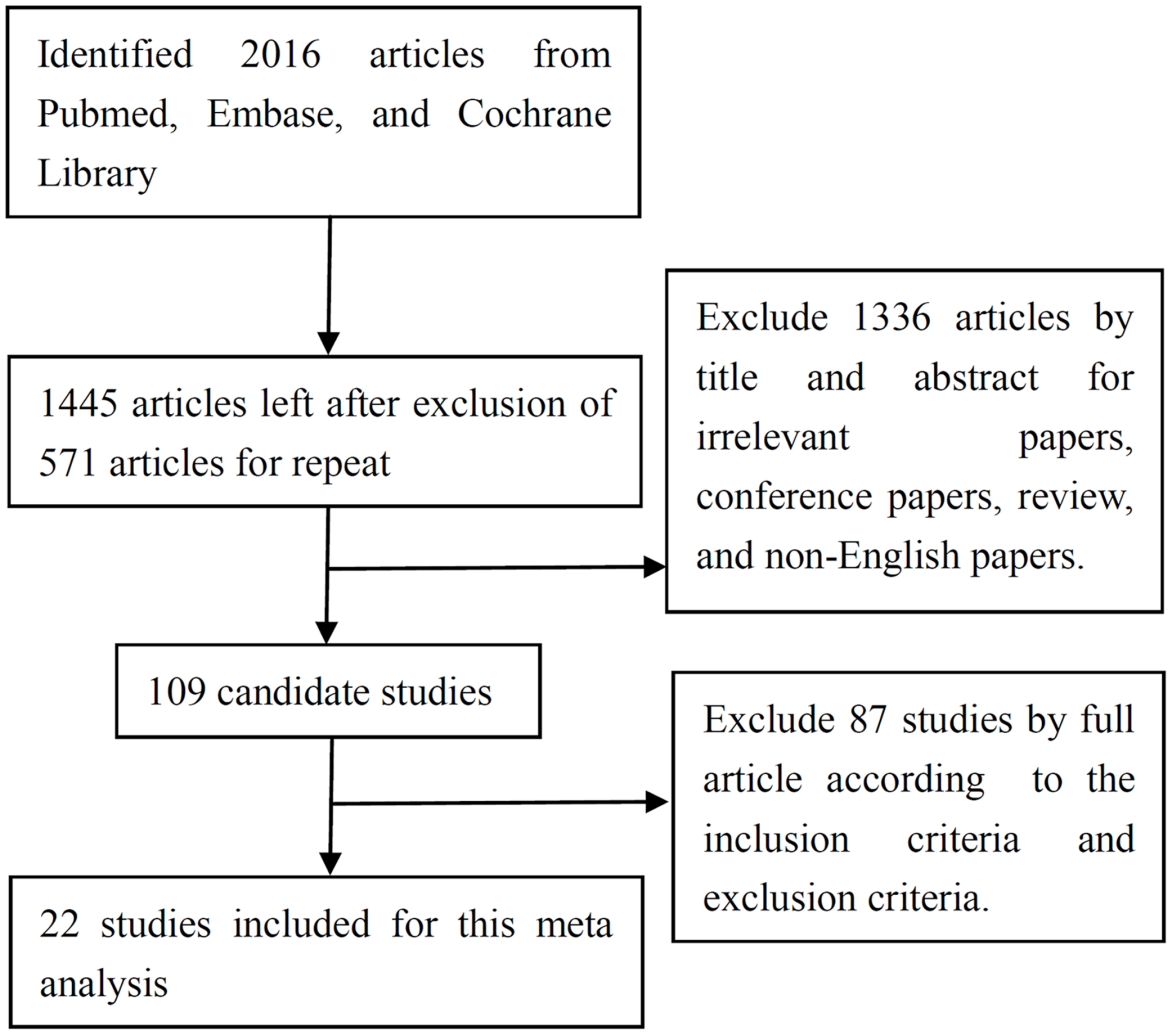
Flow chart of study selection for this meta analysis.

A total of 2016 articles were identified initially from the searched databases. Among these, 571 articles were excluded as repeated publications. Then we excluded 1336 articles as being irrelevant, conference papers, review articles, and manuscripts not published in English paper based on reading the title and abstract. Afterward, 109 candidate studies were further reviewed by reading of the full articles. In the end, 87 studies were excluded according to the inclusion and exclusion criteria, and 22 studies with 2406 patients were included for this review (Fig 1).

Among all the included studies, 2 studies assessed the frequency of *DAPK1* methylation in EC, 10 in GC, 8 in CRC, 1 in both GC and CRC, and 1 in both EC and GC. Fourteen studies were performed in Asia, five in Europe, two in South America, and one in Africa. The control group was from normal tissue in 11 studies, whereas others were from normal tissue adjacent to the tumor. All the studies were retrospective studies, and the MSPCR was used to assess the methylation of *DAPK1* in the tissue sample. The associations between *DAPK1* methylation and T stage, N stage, M stage and differentiation were presented in 9, 13, 6, and 10 studies, respectively. The characteristics of the included studies are shown in Table 1.

**Table 1:** Characteristics of the included studies.

No.	Author	Year	Country	Cancer Type	Case(cancer/ control)	Source of Control	Methylation in tumor	Methylation in Control
1	Bagci[18]	2016	Turkey(Asia)	CRC	93/14	AT	42/93	4/14
2	Laskar[19]	2015	India(Asia)	CRC	80/20	AT	27/80	6/20
3	Almeida[20]	2015	Brazil(South America)	CRC	5	AT	4/5	5/5
4	Kupčinskaitė-Noreikienė[21]	2013	Lithuanian (Europe)	GC	69	AT	33/69	32/69
5	Nomura[11]	2013	Japan(Asia)	GC	115/412	NT	95/115	201/412
6	Ye[8]	2012	China(Asia)	GC	62	AT	34/62	11/62
7	Li [22]	2011	China(Asia)	EC	47	AT	22/47	6/47
8	Hu[23]	2010	China(Asia)	GC	70/30	NT	42/70	0/30
						AT	42/70	10/70
9	Lee[12]	2009	Korea(Asia)	CRC	243/148	NT	81/243	0/148
10	Zou[24]	2009	China(Asia)	GC	16/20	NT	7/16	0/20
11	Ksiaa[25]	2009	Tunisia (Africa)	GC	68/53	AT	21/68	13/53
12	Kato[26]	2008	Japan(Asia)	GC	81/43	AT	18/81	4/43
13	Kuester[10]	2007	Germany (Europe)	EC	35/20	NT	21/35	4/20
14	Mittag [9]	2006	Germany (Europe)	CRC	22/8	AT	18/22	2/8
15	Anacleto[27]	2005	Brazil(South America)	CRC	106/30	AT	21/106	0/30
16	Chan[28]	2005	China(Asia)	GC	107/23	NT	74/107	0/23
17	Schildhaus[29]	2005	Germany (Europe)	GC	7	AT	6/7	2/7
				EC	10	AT	7/10	4/10
18	Lee [30].	2004	Korea(Asia)	CRC	149/24	NT	71/149	0/24
19	Sabbioni[31]	2003	Italy(Europe)	GC	21/6	NT	19/21	2/6
				CRC	47/4	NT	35/47	0/4
20	Waki[14]	2003	Japan(Asia)	GC	93	AT	40/93	68/93
21	Yamaguchi[32]	2003	Japan(Asia)	CRC	122/10	NT	67/122	0/10
22	To[33]	2002	China(Asia)	GC	31/10	NT	22/31	0/10

NT: normal tissue

AT: normal tissue adjacent to the tumor

### Association between *DAPK1* methylation and gastrointestinal cancer

**Fig 2:**
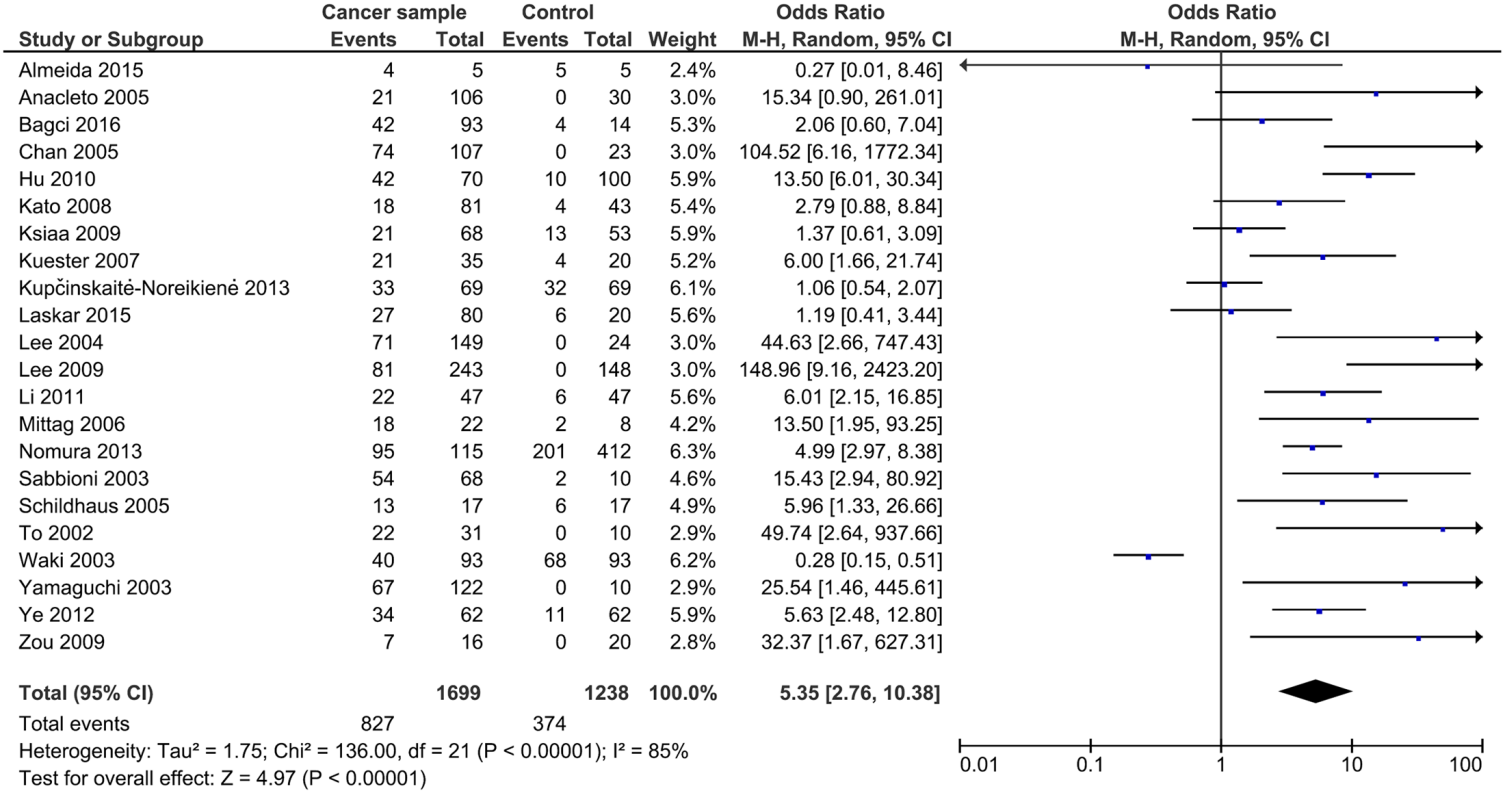
*DAPK1* methylation and the risk of gastrointestinal cancer.

**Fig 3:**
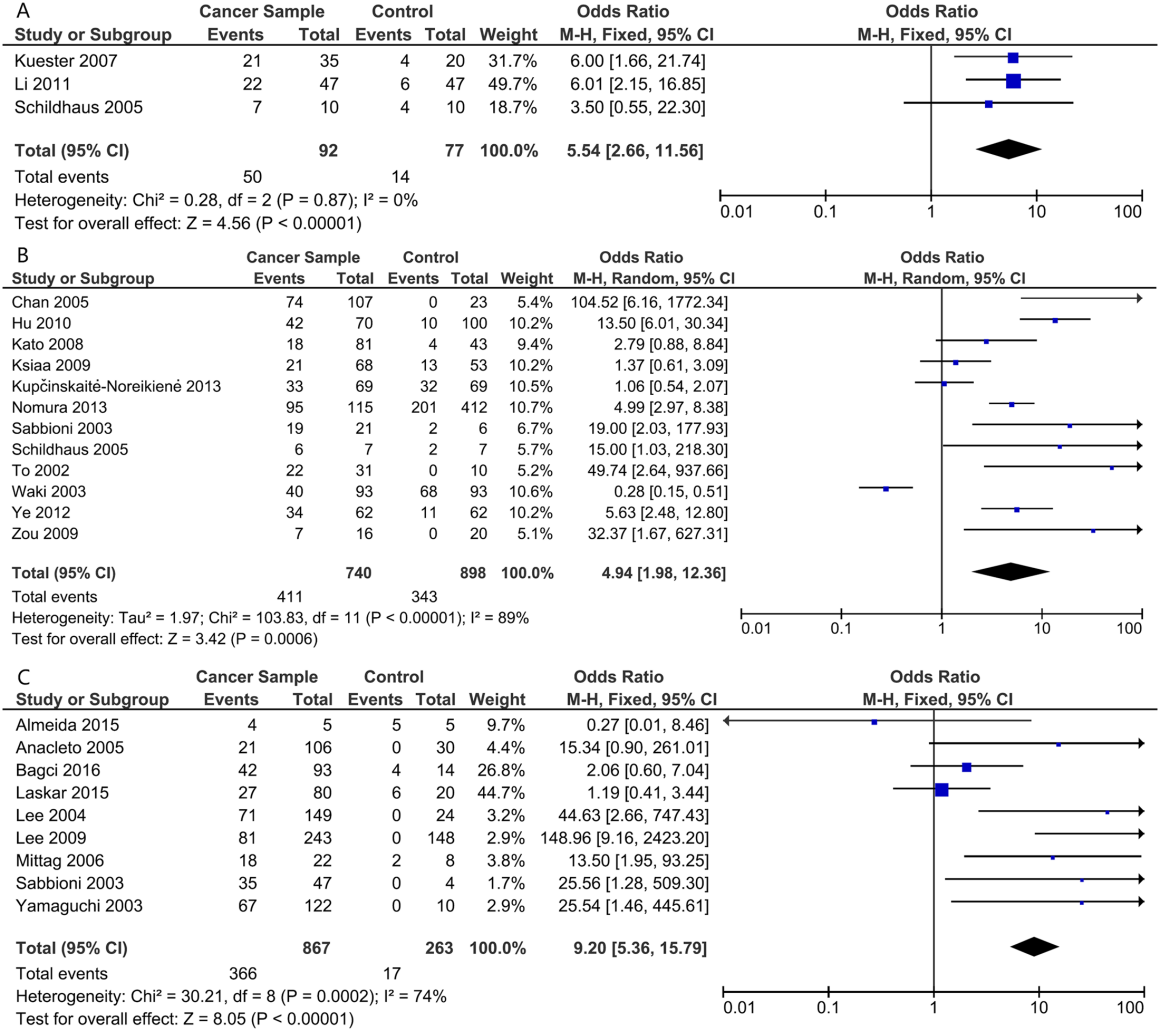
*DAPK1* methylation and the risk of different type of gastrointestinal cancer: A. esophageal cancer (EC); B. gastric cancer (GC); and C. colorectal cancer (CRC).

**Fig 4:**
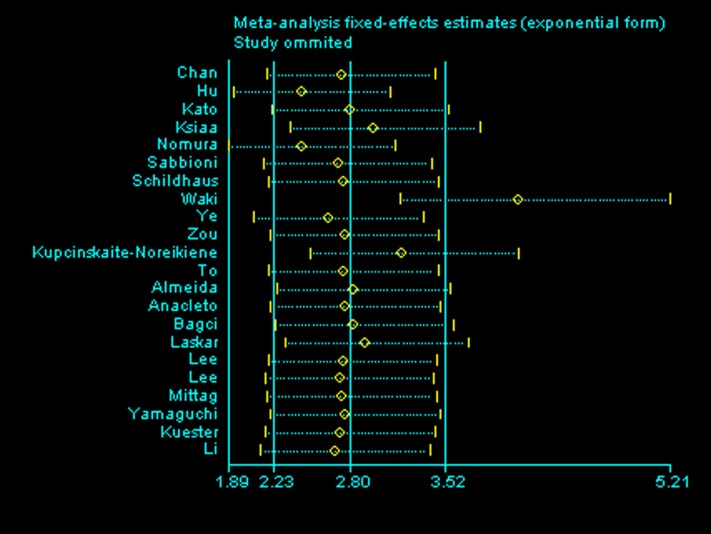
Sensitivity analysis.

Generally, the methylation of *DAPK1* was positively related to the risk of gastrointestinal cancer, with a pooled OR of 5.35 (95%CI: 2.76–10.38, P<0.00001) using the random effects model due to high heterogeneity (I^2^ = 85%, P<0.00001; Fig 2). The association was more obvious in CRC (OR = 9.20, 95%CI: 5.36–15.79, P<0.00001, fixed effects model; Fig 3). Meanwhile, the ORs were 5.54 in EC (95%CI:2.66–11.56, P<0.00001, fixed effects model), and 4.94 in GC (95%CI: 1.98–12.36, P = 0.006, random effects model; Fig 3). To find the source of heterogeneity, a sensitivity analysis was applied. As shown in Fig 4, the study conducted by Waki et al.[14] could affect the result remarkably (Fig 4). After omitting this study, the I^2^ decreased and the OR increased in both the pooled analysis (I^2^ = 72%, OR = 5.40, 95%CI: 4.30–6.78, P<0.00001, fixed effects model; S1 Fig) and GC analysis (I^2^ = 78%, OR = 5.93, 95%CI: 2.84–12.38, P<0.00001, random effects model; S2 Fig). Then subgroup analysis according to the source of the control group, geographic area, and sample size of cases were applied to further analyze the source of heterogeneity. The heterogeneity decreased most significantly in the subgroup of studies with a sample size of cases was less than 60 (I^2^ = 12% in pooled analysis). Also, in the subgroup of studies that took normal tissue as a control group, the I^2^ was lower (I^2^ = 61%) and the OR greater (OR = 12.94, 95%CI: 8.65–19.36, P<0.00001, fixed effects model). In addition, analysis in Asian patients produced a significantly increased OR (OR = 7.64, 95%CI: 2.89–20.20, P<0.0001; Table 2)

**Table 2:** Subgroup analysis of studies reporting on the association of *DAPK1* methylation and gastrointestinal cancer.

		Source of the control group		Geographic area		Sample size of case group	
		Normal tissue subgroup	Normal tissue adjacent to the tumor	Asian subgroup	Non-Asian subgroup	>60	≤60
**Overall**	Study(n)	10	12	13	8	14	7
	OR (95%CI)	12.94 (8.65, 19.36)	2.92 (2.19, 3.90)	7.74 (5.78, 10.36)	2.40 (1.62, 3.55)	5.50 (2.77, 10.92)	7.37 (4.08, 13.33)
	Model	Fixed	Fixed	Fixed	Fixed	Random	Fixed
	I^2^	61%	67%	67%	68%	79%	12%
	P	<0.00001	<0.00001	<0.00001	<0.0001	<0.00001	<0.00001
**EC**	Study(n)	1	2	1	2	-	3
	OR (95%CI)	6.00 (1.66, 21.74)	5.33 (2.17, 13.05)	6.01 (2.15, 16.85)	5.07 (1.77, 14.52)	-	5.54 (2.66, 11.56)
	Model	Fixed	Fixed	-	Fixed	-	Fixed
	I^2^	-	0%	-	0%	-	0%
	P	0.006	0.0003	0.0006	0.002	-	<0.00001
**GC**	Study(n)	6	6	7	4	7	4
	OR (95%CI)	9.04 (5.75, 14.21)	3.21 (1.39, 7.40)	7.26 (5.12, 10.29)	1.60 (1.01, 2.54)	4.12 (1.85, 9.21)	27.54 (7.23, 104.86)
	Model	Fixed	Random	Fixed	Fixed	Random	Fixed
	I^2^	59%	78%	55%	68%	84%	0%
	P	<0.00001	0.006	<0.00001	0.05	0.0006	<0.00001
**CRC**	Study(n)	4	5	5	4	6	3
	OR (95%CI)	64.96 (15.20, 277.62)	2.64 (0.84, 8.25)	10.18 (1.25, 83.19)	8.36 (2.55, 27.43)	10.53 (1.67, 66.37)	6.34 (1.80, 22.42)
	Model	Fixed	Fixed	Random	Fixed	Random	Fixed
	I^2^	0%	51%	85%	37%	81%	57%
	P	<0.00001	0.09	0.03	0.0005	0.01	0.004

### Relationship between *DAPK1* methylation and clinicopathological features of gastrointestinal cancer

**Fig 5:**
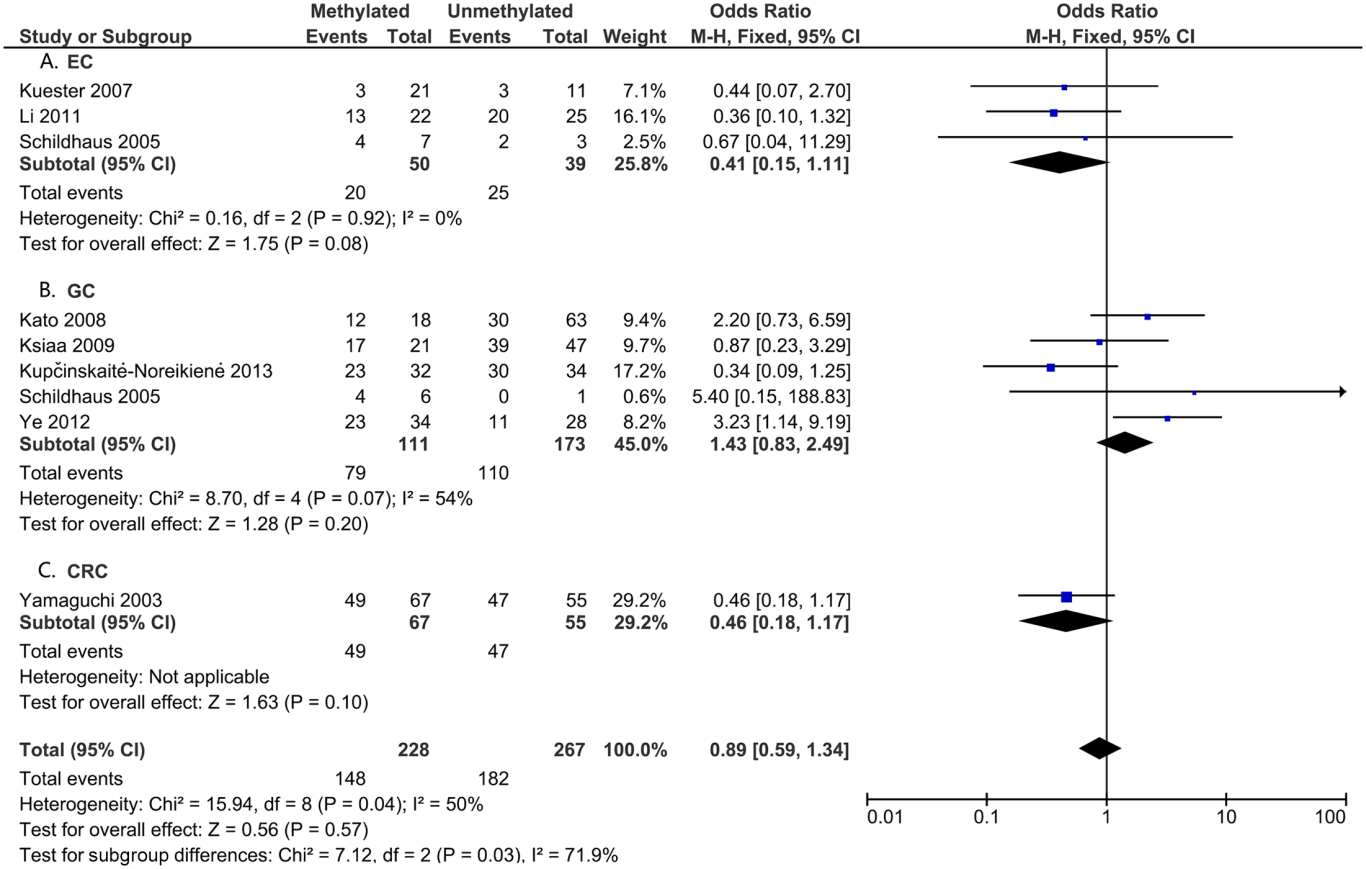
*DAPK1* methylation and T stage (T3+T4 vs.T1+T2) in: A. EC; B. GC; and C. CRC.

**Fig 6:**
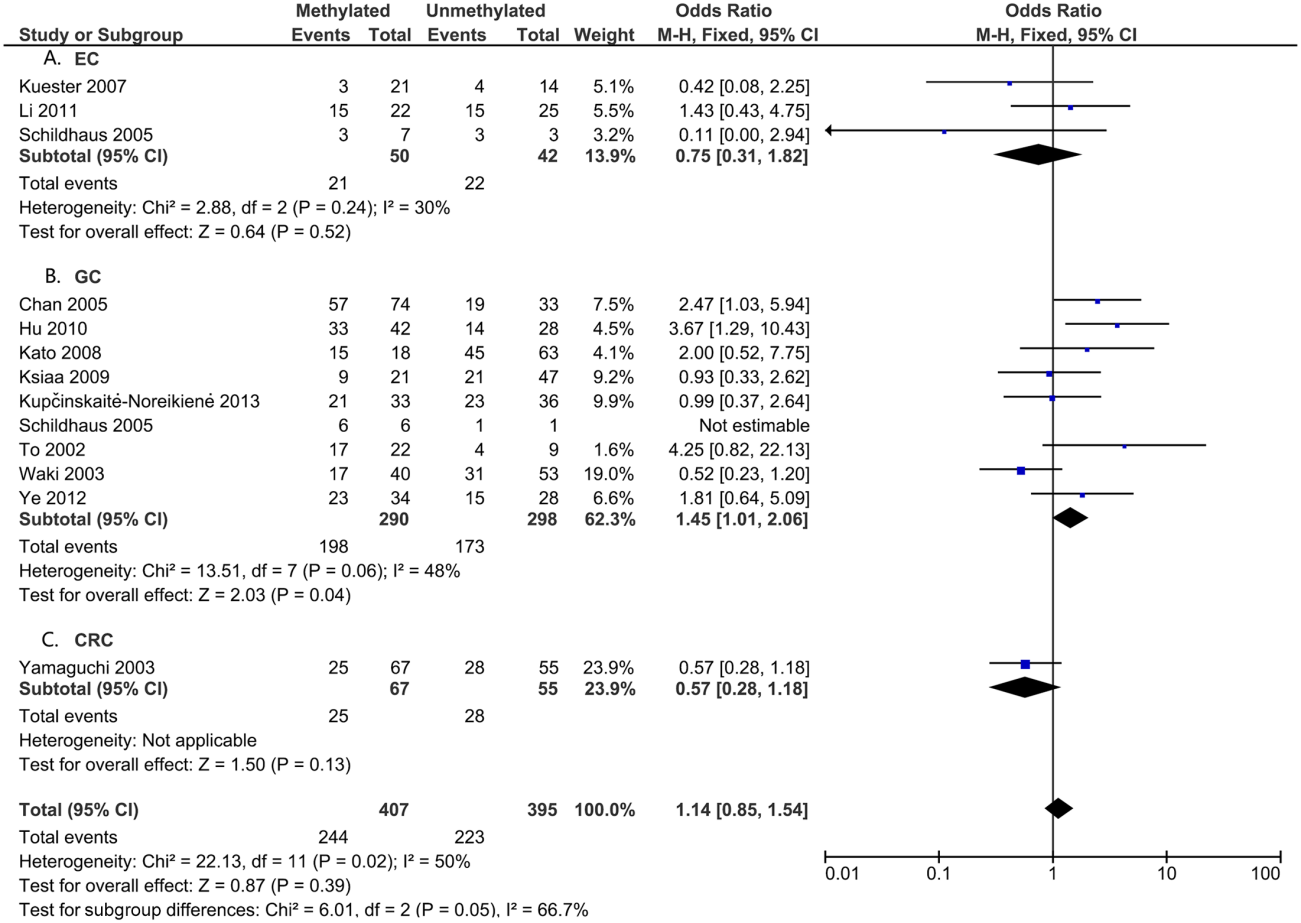
*DAPK1* methylation and N stage (positive vs. negative) in: A. EC; B. GC; and C. CRC.

**Fig 7:**
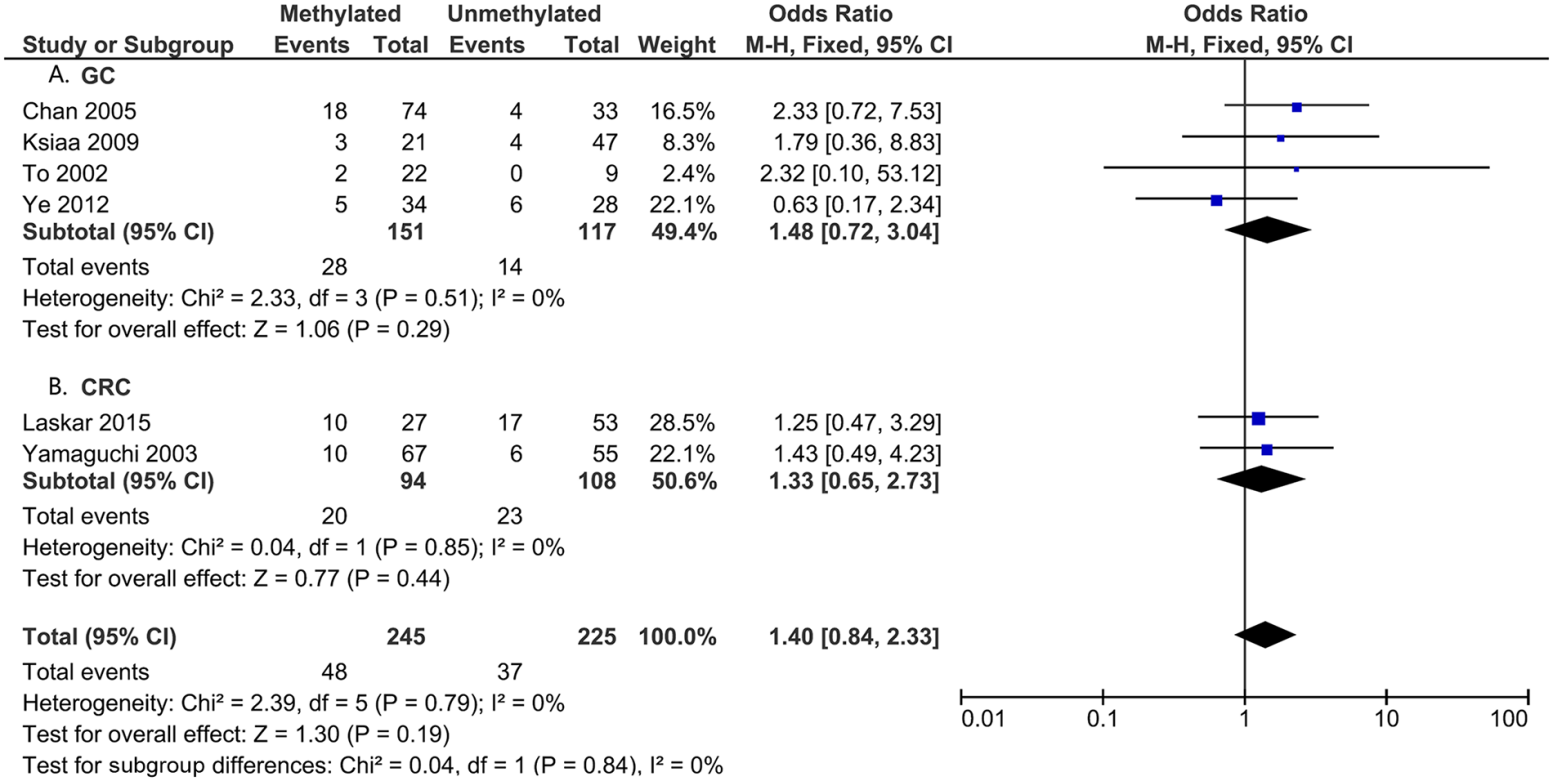
*DAPK1* methylation and M stage (M1 vs. M0): A. GC; and B. CRC.

**Fig 8:**
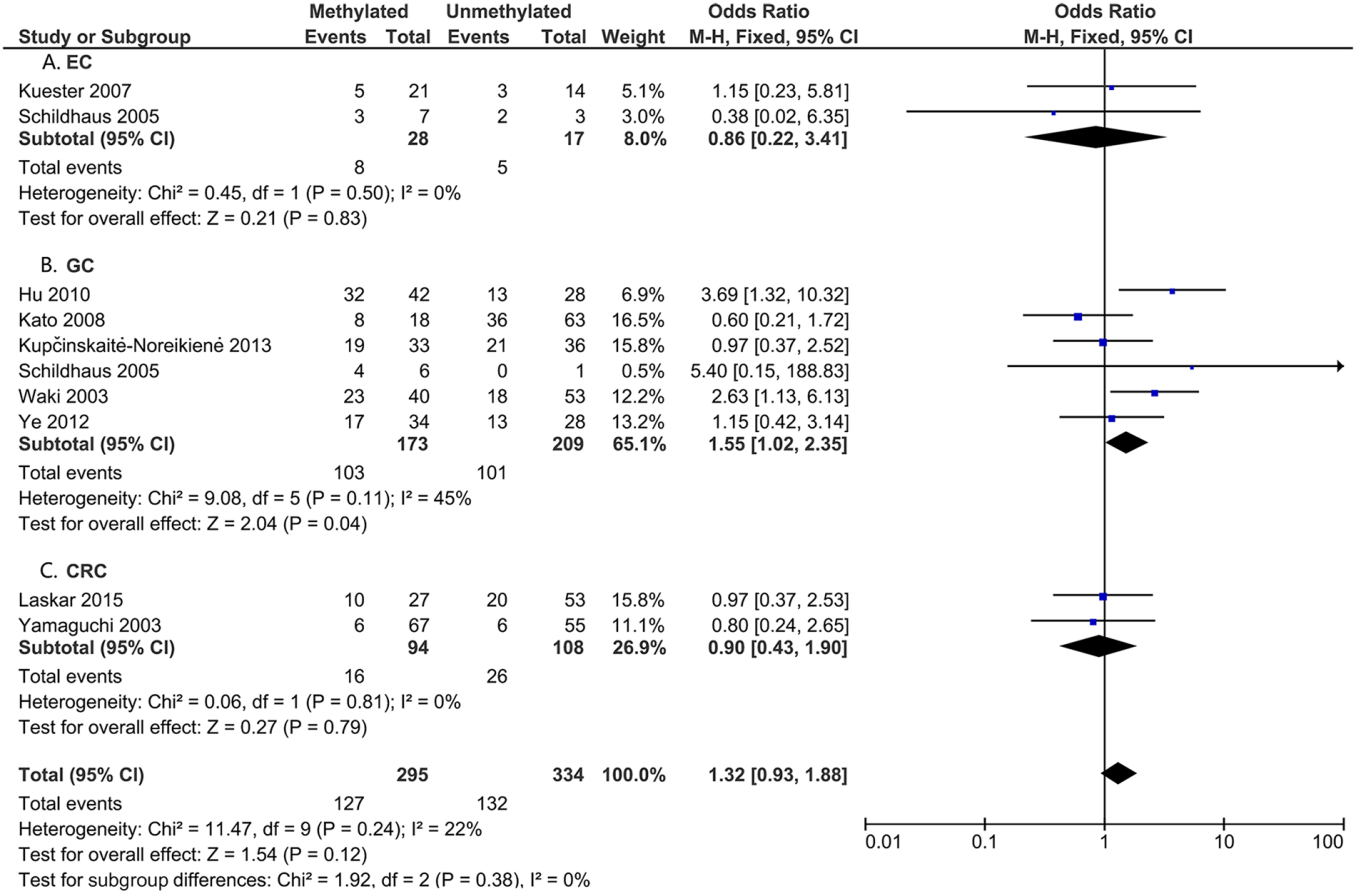
*DAPK1* methylation and cancer differentiation (G3 vs. G1+G2) in: A. EC; B. GC; and C. CRC.

To analyze the role of *DAPK1* in the pathogenesis of gastrointestinal cancer, the correlations between *DAPK1* methylation and clinicopathological features were assessed (Figs 5–8). As is shown in Fig 5, *DAPK1* methylation was not correlated with the T stage of gastrointestinal cancer (T3+T4 vs. T1+T2, OR = 0.89, 95%CI:0.59–1.34, P = 0.57, fixed effects model), nor with that of EC, GC, or CRC (Fig 5). As for N stage, *DAPK1* methylation was positively related to the N stage of GC (positive vs. negative, OR = 1.45, 95%CI: 1.01–2.06, P = 0.04, fixed effects model), but not that of gastrointestinal cancer, nor EC or CRC (Fig 6). In addition, no obvious association has been found between the methylation of *DAPK1* and the M stage of gastrointestinal cancer (Fig 7). Moreover, *DAPK1* methylation was associated with the poor differentiation of GC (G3 vs. G1+G2, OR = 1.55, 95%CI: 1.02–2.35, P = 0.04, fixed effects model; Fig 8). However, *DAPK1* methylation was not related to the age (>60 vs.<60, OR = 0.83, 95%CI: 0.54–1.27, P = 0.40, fixed effects model) or gender (male vs. female, OR = 0.48, 95%CI: 0.16–1.44, P = 0.19, fixed effects model) of gastrointestinal cancer patients (Table 3). Also, it was not correlated with the Lauren Classification of GC (intestinal vs. diffuse, OR = 1.12, 95%CI: 0.71–1.77, P = 0.63; Table 3).

**Table 3:** Associations between *DAPK1* methylation and the clinicopathological features of gastrointestinal cancer.

		Age (>60 vs. <60)	Gender (Male vs. Female)	Lauren Classification (intestinal vs. diffuse)	Asian T stage (T3+T4 vs.T1+T2)	Asian N stage (positive vs. negative)	Asia M stage (M1 vs. M0)	Asia Differentiation (G3 vs. G1+G2)
**Overall**	**Study(n)**	9	3	-	4	8	5	6
	**OR** **(95%CI)**	0.83 (0.54, 1.27)	0.48 (0.16, 1.44)	-	1.06 (0.64, 1.74)	1.29 (0.91, 1.81)	1.37 (0.80, 2.34)	1.41 (0.95, 2.10)
	**Model**	Fixed	Fixed	-	Fixed	Fixed	Fixed	Fixed
	**I**^**2**^	0%	0%	-	75%	61%	0%	48%
	**P**	0.40	0.19	-	0.83	0.15	0.25	0.09
**EC**	**Study(n)**	3	9	-	1	1	-	-
	**OR** **(95%CI)**	0.69 (0.29, 1.67)	1.16 (0.81, 1.68)	-	0.36 (0.10, 1.32)	1.43 (0.43, 4.75)	-	-
	**Model**	Fixed	Fixed	-	Fixed	Fixed	-	-
	**I**^**2**^	0%	0%	-	-	-	-	-
	**P**	0.41	0.42	-	0.12	0.56	-	-
**GC**	**Study(n)**	5	3	5	2	6	3	4
	**OR** **(95%CI)**	0.88 (0.54, 1.45)	1.01 (0.57, 1.79)	1.12 (0.71, 1.77)	2.68 (1.26, 5.72)	1.66 (1.10, 2.51)	1.41 (0.63, 3.16)	1.69 (1.06, 2.72)
	**Model**	Fixed	Fixed	Fixed	Fixed	Fixed	Fixed	Fixed
	**I**^**2**^	15%	0%	24%	0%	57%	11%	60%
	**P**	0.62	0.98	0.63	0.01	0.02	0.40	0.03
**CRC**	**Study(n)**	1	3	-	1	1	2	2
	**OR** **(95%CI)**	0.87 (0.07,10.42)	1.01 (0.57, 1.79)	-	0.46 (0.18, 1.17)	0.57 (0.28, 1.18)	1.33 (0.65, 2.73)	0.90 (0.43, 1.90)
	**Model**	Fixed	Fixed	-	Fixed	Fixed	Fixed	Fixed
	**I**^**2**^	-	0%		-	-	0%	0%
	**P**	0.91	0.98		0.10	0.13	0.44	0.79

Since the relationship between *DAPK1* methylation and gastrointestinal cancer was stronger in Asian patients, further analysis was performed in the subgroup of Asian patients to reveal the association between *DAPK1* methylation and the clinicopathological features of gastrointestinal cancer. A closer association was revealed between *DAPK1* methylation and the T stage, N stage, and differentiation of GC, for which the ORs were 2.68 (T3+T4 vs.T1+T2, 95%CI: 1.26–5.72, fixed effects model), 1.66 (positive vs. negative 95%CI: 1.10–2.51, fixed effects model), and 1.69 (G3 vs. G1+G2, 95%CI: 1.06–2.72), respectively (Table 3). However, the associations between *DAPK1* methylation and clinicopathological features were not significant in the overall analysis (Table 3).

The data for 5-year OS/DFS rates were insufficient to conduct a survival analysis.

### Publication bias

**Fig 9:**
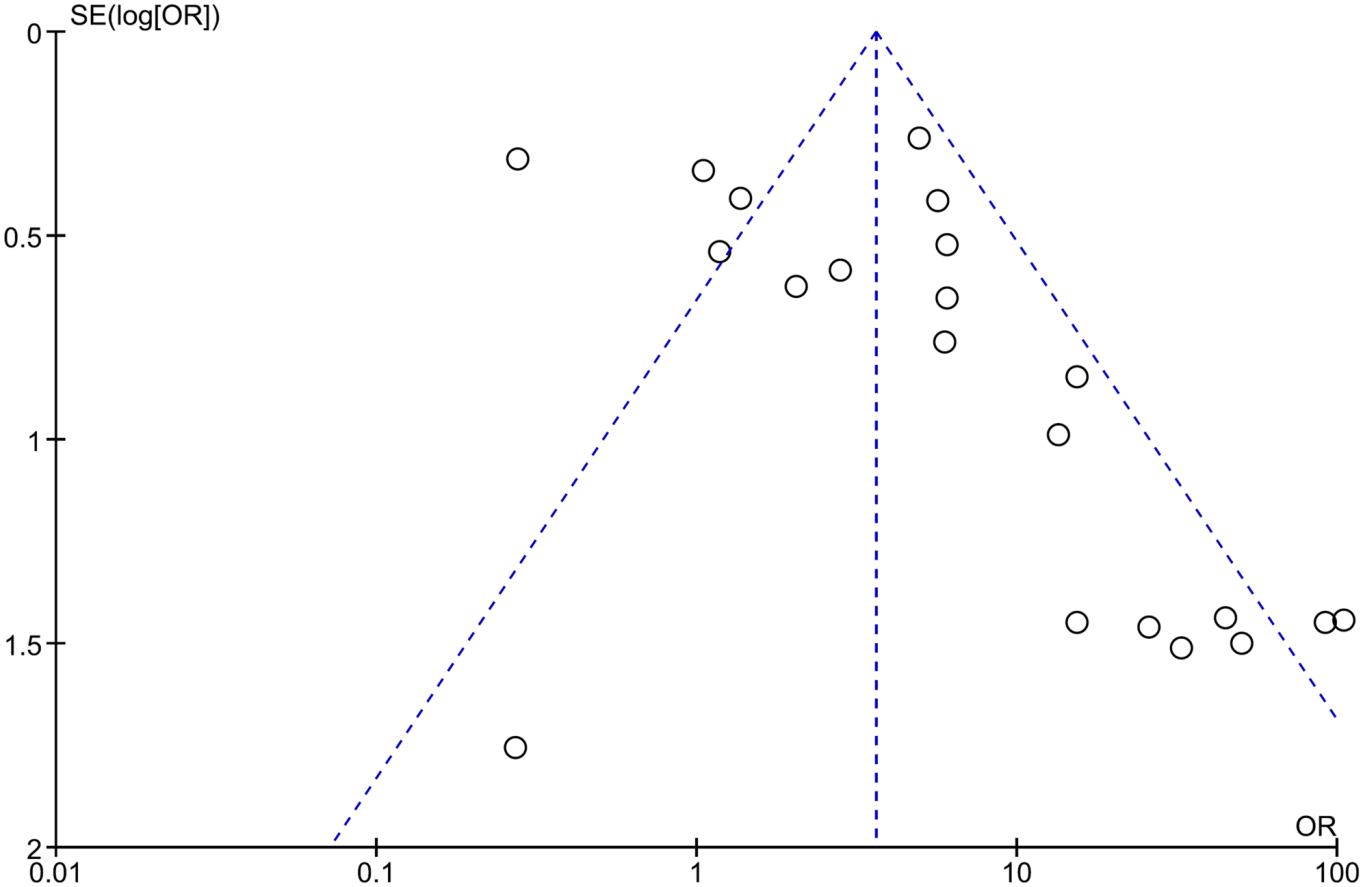
Funnel plot of the result of pooled analysis.

The shape of the generated funnel plot seemed asymmetrical in the pooled analysis (Fig 9). In addition, P values<0.05 were calculated for Egger’s tests and Begg’s tests in the overall analysis, which suggests the existence of publication bias (Table 4). However, in the analysis of the association between *DAPK1* methylation and the clinicopathological features of gastrointestinal cancer, the P values on Egger’s tests and Begg’s tests were greater than 0.05, except for the Egger’s test result for the T stage of EC (P = 0.007; Table 4 and S2 Table).

**Table 4:** Analysis of publication bias among included studies.

		Study(n)	P value of Egger’s test	P value of Begg’s test
**Pooled analysis**	Overall	22	0.024	0.032
	EC	3	0.259	1.000
	GC	12	0.141	0.244
	CRC	9	0.040	0.005
**T stage** **(T3+T4 vs. T1+T2)**	Overall	9	0.992	0.251
	EC	3	0.007	1.000
	GC	5	0.968	1.000
**N stage** **(positive vs. negative)**	Overall	13	0.806	0.373
	EC	3	0.233	1.000
	GC	9	0.191	1.000
**Metastasis** **(M1 vs. M0)**	Overall	6	0.730	0.707
	GC	4	0.863	0.734
	CRC	2	-	1.000
**Differentiation** **(G3 vs. G1+G2)**	Overall	9	0.723	1.000
	EC	2	-	1.000
	GC	6	0.823	1.000
	CRC	2	-	1.000
**Age** **(>60 vs. <60)**	Overall	8	0.491	0.446
**Gender** **(Male vs. Female)**	Overall	14	0.993	0.661
**Lauren Classification** **(intestinal vs. diffuse)**	GC	5	0.618	0.462

## Discussion

Consistent with the goal of precision medicine, molecular pathological epidemiology (MPE) based on molecular classification of disease is becoming increasingly attractive[34]. This approach can discover molecular biomarkers, identify relevant subtypes, and establish the relationship between the risk factors with specific subtype[35]. Various environmental and lifestyle factors such as one-carbon metabolism, cigarette smoking, and diet could be associated with aberrant DNA methylation, which was found to be an important biomarker and novel target for treatment in various cancers[36]. Abnormal methylation of the promoter is a critical mechanism for the down-regulation of genes including *DAPK1*[26].

*DAPK1*, as a classical anti-oncogene, has been demonstrated to play an important role in the development, progression and metastasis of tumors[7]. Down-regulation of *DAPK1* expression has been correlated with the severity of malignancy and lymph node metastasis in various cancers including lung cancer[37], urinary tract carcinoma[38], and esophageal squamous cell carcinoma[39]. It has been shown that *DAPK1* can influence cell survival and apoptosis by activating the mammalian target of rapamycin complex1 (mToRC1)[40]. Up-regulation of *DAPK1* alleviates the malignant behavior of pancreatic carcinoma through the PI3K/Akt and ERK pathway[41]. In addition, *DAPK1* is involved in activating the mTOR pathway by breaking the TSC1/TSC2 complex in the p53-mutant triple receptor–negative breast cancer[42].

Hypermethylation of *DAPK1* has been found to be involved in head and neck cancers[43], papillary thyroid cancer[44], and even brain metastases of various solid tumors[45]. Recently, several studies have investigated the roles of *DAPK1* methylation in cervical cancer[46], lung cancer[47] and GC[48]. However, a systematical analysis of its role in gastrointestinal cancer has not been reported. Therefore, the present study was needed to uncover the potential value of *DAPK1* methylation in the diagnosis and pathogenesis of gastrointestinal cancer.

The pooled OR indicated that *DAPK1* methylation was positively correlated with the risk of gastrointestinal cancer, which suggests the potential value of *DAPK1* methylation in the diagnosis of gastrointestinal cancer, especially in CRC. In addition, in the subgroup analysis of studies that used normal tissue as the control group, a tighter relationship was demonstrated. The dissimilar results for the different sources for the control group suggested that the degree of *DAPK1* methylation in the normal tissue adjacent to tumor tissue was higher than that in normal tissues. These findings were consist with previous results showing that *DAPK1* methylation is significantly related to the risk of precancerous lesions such as intestinal metaplasia (IM) [49] and Barrett’s metaplasia[10]. Moreover, the frequency of *DAPK1* methylation was shown to gradually increased from precancerous lesions to cancer[10, 24]. Therefore, detection of *DAPK1* methylation could be used for the early diagnosis of gastrointestinal cancer. In addition, the association of *DAPK1* methylation with the risk of gastrointestinal cancer was most notable in Asian patients and in CRC patients, which suggests that the pathogenic role of *DAPK1* methylation in different geographical regions and tumor locations of gastrointestinal cancer vary.

Furthermore, we investigated the associations between the frequency of *DAPK1* methylation and the clinicopathological features of gastrointestinal cancer. Our results showed that *DAPK1* methylation was unrelated to cancer differentiation, T stage, N stage, or M stage in gastrointestinal cancer. Such results indicated that *DAPK1* methylation could promote the carcinogenesis process but not the processes of invasion and metastasis[32]. When stratified by location, *DAPK1* methylation was positively correlated with lymph node metastasis and poor differentiation in GC, moreover the correlation was more significant among Asian patients, which suggests that *DAPK1* methylation was involved in the metastasis of GC in Asian patients. In addition, it is more accurate to assess the prognosis of gastrointestinal cancer by combining analysis of *DAPK1* and other genes, because the number of methylated gene gradually increases from 0.12 and 0.8 in adjacent normal tissues to 3.3 and 2.5 in GC[25] and EC tissues[22], respectively. Although the frequency of *DAPK1* methylation was found to increase with ages[50], we found that methylation of *DAPK1* was not correlated with age in gastrointestinal cancer patients.

The survival analysis showed that *DAPK1* methylation was correlated with the susceptibility of recurrence, metastasis and disease-related death (67.6% in methylated group vs. 41.9% in unmethylated group) in GC[28]. However, in other studies, *DAPK1* methylation was not associated with OS in GC [14] or EC[51]. Such disagreement suggests that more studies are needed for more conclusive survival analysis.

Inevitably, there are some limitations in this meta analysis. First, heterogeneity existed in some analyses, though it could be alleviated by the sensitivity analysis and subgroup analysis according to the potential heterogeneous factors, such as the source of the control group, geographic area, and tumor location. To better analyze the association between *DAPK1* methylation and gastrointestinal cancer, a more precise method like the qMSP should be used in future studies to distinguish the degree of the methylation [52]. In addition, potential publication bias is inevitable, and the existence of publication bias in the overall analysis may reduce the power and accuracy of the relationship between *DAPK1* methylation and gastrointestinal cancer. Last but not least, the association between *DAPK1* methylation and the survival of patients could not be estimated due to an insufficient amount of related data. The above limitations may partially influence the significance of *DAPK1* methylation and the clinicopathological analyses. Therefore, larger prospective studies are needed to validate our results.

In summary, the findings of this meta-analysis indicate that the methylation of *DAPK1* may be valuable biomarker in the diagnosis and the tumorgenesis of gastrointestinal cancer. However, *DAPK1* methylation was not correlated with the clinicopathological features of gastrointestinal cancer, but was associated with the N stage and cancer differentiation of GC. Thus, further studies of *DAPK1* and its potential role in the progression of gastrointestinal cancer are needed.

## Supporting information

S1 Fig Pooled analysis of *DAPK1* methylation and gastrointestinal cancer after omitting the heterogeneous study (Waki et al 2003). (TIF)

S2 Fig Association of *DAPK1* methylation and GC after omitting the heterogeneous study (Waki et al 2003). (TIF)

S1 Table PRISMA 2009 checklist. (DOC)

S2 Table Publication bias of subgroup analysis. (DOCX)
