# Supplementary figures and images for: Correlation of DAPK1 methylation and the risk of gastrointestinal cancer: A systematic review and meta-analysis
Source: PLoS One. 2017 Sep 21;12(9):e0184959. doi: 10.1371/journal.pone.0184959 (PMC5608298; doi:10.1371/journal.pone.0184959)

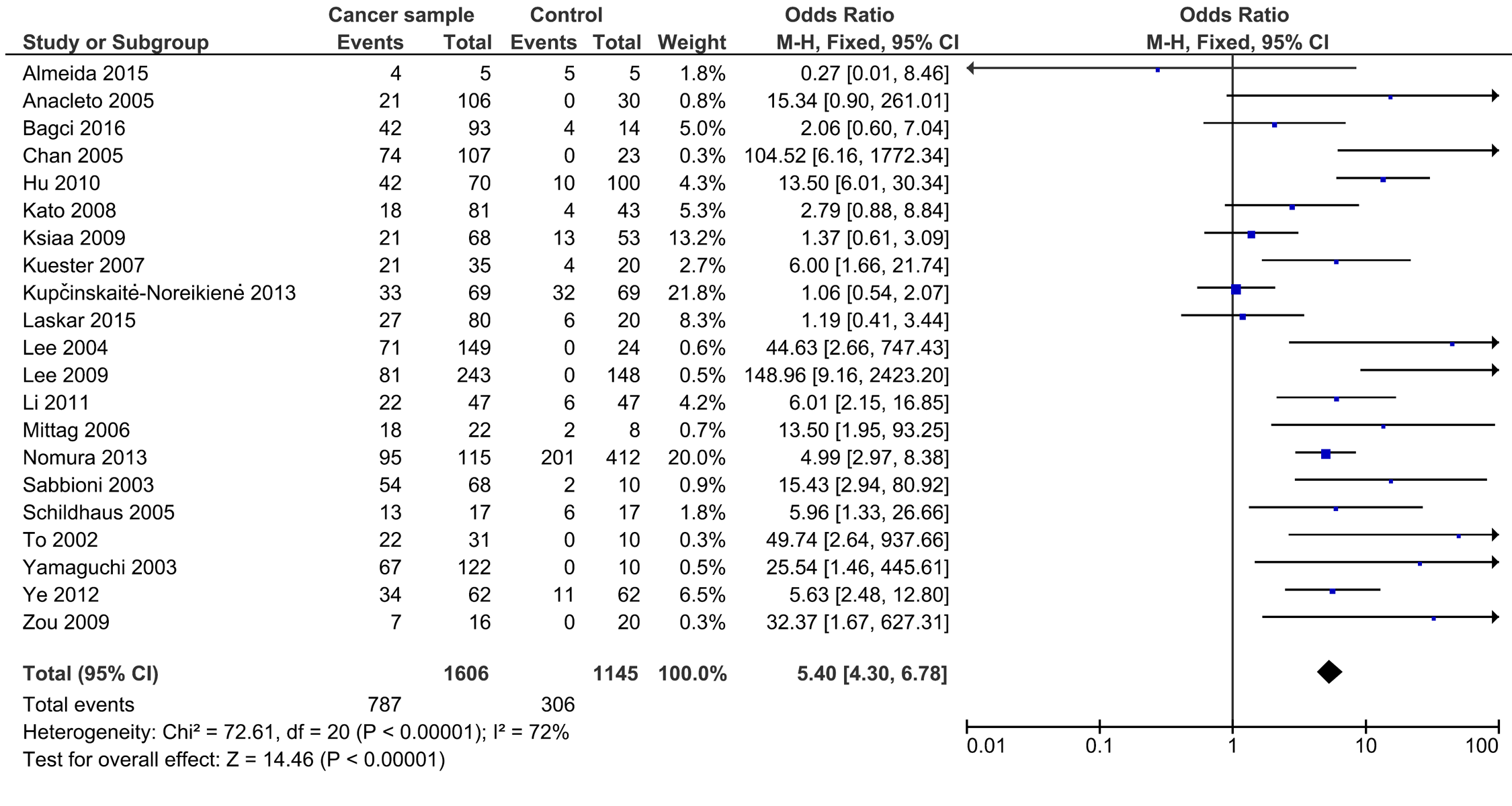

Supplement: S1 Fig — (TIF) [file pone.0184959.s001.tif]

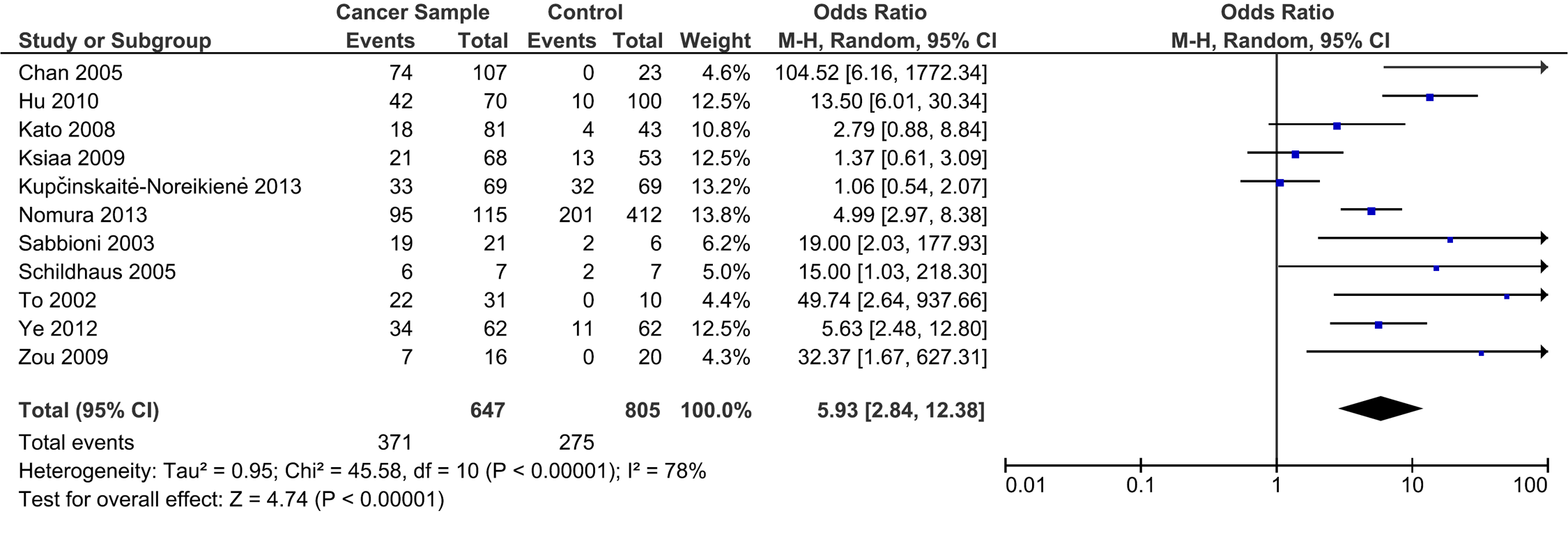

Supplement: S2 Fig — (TIF) [file pone.0184959.s002.tif]
