# Supplementary material for: Correlation of DAPK1 methylation and the risk of gastrointestinal cancer: A systematic review and meta-analysis
Source: PLoS One. 2017 Sep 21;12(9):e0184959. doi: 10.1371/journal.pone.0184959 (PMC5608298; doi:10.1371/journal.pone.0184959)
Supplement: S2 Table — (DOCX) [file pone.0184959.s004.docx]

|  |  | Studies(n) | P value of Egger test | P value of Begg test |
| --- | --- | --- | --- | --- |
| Normal tissue subgroup | Overall | 11 | 0.000 | 0.005 |
|  | EC | 1 | - | - |
|  | GC | 6 | 0.005 | 0.133 |
|  | CRC | 4 | 0.289 | 0.089 |
| Normal tissue adjacent to the tumor subgroup | Overall | 12 | 0.499 | 0.193 |
|  | EC | 2 | - | 1.000 |
|  | GC | 6 | 0.420 | 0.452 |
|  | CRC | 5 | 0.574 | 0.086 |
| Asian subgroup | Overall | 14 | 0.035 | 0.228 |
|  | EC | 1 | - | - |
|  | GC | 8 | 0.208 | 1.000 |
|  | CRC | 5 | 0.006 | 0.027 |
| Non-Asian subgroup | Overall | 8 | 0.091 | 0.063 |
|  | EC | 2 | - | 1.000 |
|  | GC | 4 | 0.002 | 0.308 |
|  | CRC | 4 | 0.480 | 0.734 |
| Subgroup of sample size of case group >60 | Overall | 14 | 0.094 | 0.037 |
|  | EC | 0 | - | - |
|  | GC | 7 | 0.542 | 0.230 |
|  | CRC | 6 | 0.003 | 0.133 |
| Subgroup of sample size of case group ≤60 | Overall | 7 | 0.742 | 0.133 |
|  | EC | 3 | 0.259 | 1.000 |
|  | GC | 4 | 0.334 | 0.089 |
|  | CRC | 3 | 0.589 | 1.000 |
| Asian T stage  (T3+T4 vs. T1+T2) | Overall | 4 | 0.969 | 0.089 |
|  | EC | 1 | - | - |
|  | GC | 2 | - | 1.000 |
|  | CRC | 1 | - | - |
| Asia N stage  (positive vs. negative) | Overall | 8 | 0.068 | 1.000 |
|  | EC | 1 | - | - |
|  | GC | 6 | 0.260 | 1.000 |
|  | CRC | 1 | - | - |
| Asia M stage  (M1 vs. M0) | Overall | 5 | 0.853 | 0.806 |
|  | EC | 0 | - | - |
|  | GC | 3 | 0.936 | 1.000 |
|  | CRC | 2 | - | 1.000 |
| Asia Differentiation  (G3 vs. G1+G2) | Overall | 6 | 0.282 | 0.260 |
|  | EC | 0 | - | - |
|  | GC | 4 | 0.501 | 0.308 |
|  | CRC | 2 | - | 1.000 |
